# Learning to mentalize: Exploring vulnerable parents’ experiences of change during video guidance in an infant mental health clinic

**DOI:** 10.1186/s12888-021-03398-6

**Published:** 2021-08-12

**Authors:** Indra Simhan, Kari Vik, Marius Veseth, Aslak Hjeltnes

**Affiliations:** 1 Department of Child and Adolescent Mental Health, Sorlandet Hospital, PO Box 416, N-4604 Kristiansand, Norway; 2 Faculty of Psychology, University of Bergen, Bergen, Norway

**Keywords:** Parents, Infant, Parental mental health, COPMI, Video guidance, Video feedback, Marte Meo, Thematic analysis, Parent development interview

## Abstract

**Background:**

Interventions that promote infant mental health face challenges when applied for parents who struggle with psychosocial and psychological burdens. Video-based guidance using the Marte Meo method is used in specialized clinical settings with high-risk families to improve parent-child interaction, parental sensitivity and mentalizing. However, knowledge about the lifeworlds of these parents and their experiences of the therapeutic process during video guidance is limited.

**Aim:**

This qualitative study explores how parents in an infant mental health outpatient clinic who had difficulties mentalizing and maintaining an emotional connection with their infants experienced the change process during Marte Meo video guidance.

**Methods:**

We identified a strategic sample of parents with difficulties mentalizing and maintaining an emotional connection with their infants through the Parent Development Interview. Twelve parents received video guidance and were afterwards interviewed in-depth. The research interviews were qualitatively analysed via a team-based reflexive thematic analysis.

**Result:**

We identified four themes: a) feeling inadequate or disconnected as a parent; b) discovering the infant as a relating and intentional person; c) becoming more agentic and interconnected; and d) still feeling challenged by personal mental health issues.

**Conclusion:**

Parents described positive changes in their interactions, in mentalizing their infants, the relationship and themselves as parents, in their experiences of self-efficacy and on a representational level. They also described increased confidence and improved coping despite ongoing personal mental health challenges. The findings suggest that video guidance using the Marte Meo method can be a critical intervention for vulnerable parents but should be coordinated with parents’ primary treatments when complex parental mental health issues are involved.

## Background

What is it like to engage in video guidance focusing on your their parenting skills when you are struggling to understand and relate to your own infant and are burdened by mental health problems? How do parents with mentalization difficulties experience their own change processes, and what can we learn about video guidance from these parents’ experiences? In this study, we present an investigation of video guidance in an infant mental health outpatient clinic for parents with difficulties mentalizing their infants and maintaining an emotional connection with them.

A parent’s capacity to mentalize the infant’s experiences, that is, to see the infant as an intentional being, to understand overt behaviours in terms of the infant’s underlying mental states, and to regulate the infant’s emotions, is associated with parental sensitivity and secure attachment [1]. Enhancing parents’ mentalizing and promoting sensitive, attuned caregiving is therefore a central aim of parent-infant interventions [2]. Among interventions, video guidance approaches that use film clips of actual interactions to guide parents are a versatile group of methods for both home visits and clinical settings [3]. The positive effects of video guidance for parent-child dyads have been comprehensively documented and to some extent conceptualized [4, 5]. Marte Meo video guidance [6] and its manualized variant, video guidance of parent-infant interaction (VIPI) [7], use videos of everyday situations between parents and infants. For the review sessions with caregivers, therapists choose clips showing developmentally supportive interactions or an interactional opportunity for them and makes use of micro-interaction sequences and stills. Marte Meo thus has a solution-focused stance, omitting material that shows a negative or ineffectual interaction [6, 8]. Research on Marte Meo has hitherto been mainly based on community samples. Studies of mothers with postnatal depressive symptoms have shown how Marte Meo enhanced their vitality and invited them to self-reflection and other-centeredness in the presence of sensitive and attuned therapists. These studies have conceptually linked Marte Meo to Stern’s theories of early communication and mentalization, especially mentalized affectivity [9, 10]. Quantitative studies have identified positive effects of Marte Meo on parental sensitivity, interaction, and infant development also within more vulnerable dyads [7, 11, 12]. However, there is a lack of research on Marte Meo in specialized clinical settings, where parents are characterized by more severe psychosocial stress and mental health problems, frequently suffering from affective disorders, substance abuse, personality disorders and other disorders [13–15]. This lack of scholarship also concerns parents’ lifeworlds, or lived experience. Often, their intuitive parenting capacity is weakened, and they have difficulties establishing and regulating their emotional connections to their infants and discovering and mentalizing the infants’ signals [16, 17]. In community samples, Marte Meo has been shown to be effective for parents with depression, with certain personality disorders [7], and with high psychosocial risks [12]. A three-case study with parents in a specialized infant clinic has suggested that the therapeutic contexts in which the videos are shown could support a positive circle of mutuality, reflexivity and self-confidence, centrally enhancing parental sensitivity [10]. More empirical knowledge is needed about how parents with challenges in key capacities of parenting experience and respond to Marte Meo video guidance during specialized infant mental health services. This knowledge would contribute to elucidating the processes involved in parenting under duress and to adapting the Marte Meo method to the requirements of this parent group. The present study is part of a larger body of data from Marte Meo in specialized settings. An analysis of vulnerable parents’ experiences of both helpful and hindering aspects of video guidance is available in (Simhan I, Vik K, Veseth M, Hjeltnes A: Like taking a magnifying glass into everyday life: vulnerable parents’ experiences with video guidance in an infant mental health clinic, submitted for publication.). It highlights the challenges and opportunities of using video guidance as a method for parents who tend to feel highly insecure, self-critical, threatened or devalued. None of the themes presented in this study were reported in our previous publication.

The first aim of the present study was to explore the lived experience of changes during video guidance for a strategic sample of clinically referred parents who had difficulties mentalizing and maintaining emotional connections with their infants. Its second aim was to develop a conceptual understanding of Marte Meo video guidance and its clinical uses for these clients. We asked the following research question: How do parents who find it difficult to mentalize or maintain an emotional connection with their infants experience the change process or a lack of changes during Marte Meo video guidance?

## Methods

To explore the lived experience of parents receiving Marte Meo video guidance in specialized mental health settings, we used open-ended in-depth interviews [18] that were subjected to an explorative, team-based reflexive thematic analysis (TA) [19, 20].

### Setting

The study was a collaboration between the Research Unit and the Infant Mental Health Team (IMHT), an outpatient service for parent-infant dyads, the Department for Child and Adolescent Mental Health at the Southern Norway Hospital Trust, Kristiansand, and the Department of Clinical Psychology at the University of Bergen, Norway. Clinicians from the IMHT assisted in the recruitment process and administered video guidance using the Marte Meo method to the participants.

### Recruitment of participants

To recruit a strategic sample of parents of infants who had been referred to the IMHT and experienced difficulties mentalizing or maintaining emotional connections with their infants, we employed criterion sampling [21], assisted by the Parent Development Interview, revised version (PDI-R). The PDI-R is a 45-item semi-structured narrative interview about parents’ representations of their children, themselves as parents, and the parent-child relationships (Slade A, Aber JL, Bresgi I, Berger B, Kaplan M. Parent Development Interview, Short Revised Version (PDI-R)). Unpublished protocol. 2004). A subset of twelve items can be rated in terms of reflective functioning (RF), an operationalized measure of mentalization manifesting in speech, or the capacity to recognize and understand internal experiences in terms of underlying mental states [22]. The sampling criterion was either a) limited RF in the PDI-R or b) explicitly stated difficulties emotionally connecting with the infant and maintaining the connection during affective stress. Based on the concept of information power [23] and considering the aim, framework and specificity of experience and the expected quality of dialogue, we targeted a sample of 10 to 15 participants.

The PDI-R was administered to 30 consenting parents where video guidance was clinically indicated for the dyads. The interviews were digitally recorded, transcribed verbatim, and then read by the first author, a certified rater, who rated the subset of items on the 11-point RF-scale from limited to high RF [24]. An overall score of 4 or less represented limited RF. Thirty percent of the PDI-R was double-coded by two certified, blinded external raters, with an intraclass correlation of 0,89 (95%-confidence interval 0.58 to 0.97) [25]. Quantitative data from the strategic sampling process were not used in the qualitative analysis.

### Strategic sample

We recruited a strategic sample of 15 parents who met criterion a) or b). Parents received Marte Meo video guidance and were afterwards interviewed in-depth about their experiences. One parent was excluded because her infant was removed from her care during the guidance, and two parents declined to participate in the research interview for personal reasons. We conducted in-depth interviews with 12 parents, including 11 mothers and one father, from 23 to 34 years of age (M = 27). All recruited dyads were referred to specialized treatment based on the risk to their infant linked to parental functioning or mental health issues. The infants were 6 males and 6 females, and infant ages ranged from 2 to 27 months (M = 9 months) at the start of the guidance. Ten parents met criterion a), limited mentalization capacity; and two met criterion b), experiencing a lack of emotional connection. Eight parents reported adverse or traumatic childhood experiences. All parents reported mental health issues, mostly of long-term or recurrent natures, including posttraumatic stress disorders, personality disorders, uni- and bipolar affective episodes, obsessive-compulsive disorders, and substance misuse disorders.

### Intervention/video guidance

Marte Meo video guidance courses [6] were tailored to each individual dyad. One of four Marte Meo therapists from the IMHT with extensive experience with parent-infant dyads administered each dyad’s guidance. Filming sessions generally took place during home visits. All parents received 3–7 guidance sessions (M = 4 sessions).

### In-depth interviews

We devised a semi-structured interview guide to assist the interviewing, asking about participants’ experiences with the course of video guidance, the therapeutic relationship, particularly helpful or hindering aspects of the guidance, and changes that might have taken place regarding their thoughts about their infants and parent-infant relationships. Some example questions were the following: ‘Is there something from the guidance that you specifically remember?’, ‘How did you experience filming?’, ‘How did you experience the therapist? Was something specific he/she did helpful or not helpful?’, and ‘Concerning your thoughts about your infant, has the guidance changed these in any way?’ We used the guide to structure the interviews but encouraged participants to pursue the topics that they found relevant. All interviews (*n* = 12) were conducted by the first author from November 2016 to June 2019, lasting between 23 and 89 min (M = 61 min). The interviews took place 2–6 months after the end of the guidance (M = 3 months). They were digitally recorded and transcribed verbatim.

### Methodology

We chose an explorative, reflexive thematic analysis (TA) as the method for an inductive, data-driven analysis [20, 26]. We aimed at a reflexive, experience-near reporting of the data and carried out the analysis through a team-based approach [19], which further strengthened the balance between closeness to the participants’ experiences and reflections of our own positions as researchers. Conceptualizing, including conceptual language, was consciously set aside during data analysis.

### Data analysis

We used NVivo 11 qualitative data analysis software [27] for technical assistance. Reflexive TA was carried out in seven steps by the first, third and last author in a collaborative process. 1) All collaborators familiarized themselves with the data and noted their first impressions and reflections about the experiences related to each interview. 2) The first author reread each interview, identifying meaning units and generating 40 initial codes. Meaning units were understood as features of the data that appeared interesting or seemed to convey meaning regarding the phenomenon. The existing codes were used across interviews only if they were considered to provide suitable descriptions. 3) The first author reported the coded meaning units back to the group, and 34 meaning patterns or subthemes and 4 main themes were formulated in a collaborative process, using the first impressions, the initial codes, and referencing back to the transcripts. 4) Themes were summarized and reviewed as a back-and-forth process between the first author and the group, maintaining the 4 main themes and formulating 21 most relevant sub-themes. 5) The first author refined the themes and wrote an analysis of each one. 6) The themes were assembled in a written form related to the research questions. 7) The research team formed a consensus on the formulation of the main thematic categories.

### Researchers

The first author, a child psychiatrist, and the second author, a sociologist, are Marte Meo video guidance therapists specializing in infant mental health. The third and fourth authors are associate professors in clinical psychology. All the authors have extensive clinical experience with psychotherapy and mental healthcare, as well as experience with qualitative research on a range of topics in mental health.

### Ethics

The study was approved by the Norwegian Regional Council for Research Ethics. The clinicians at the IMHT gave oral and written information about the study to the suitable parents and obtained all participants’ written informed consent for their participation and the study’s publication. Participants could withdraw from the study without consequences for their treatment. The researchers were aware of the vulnerable positions of both the parents and infants involved in the research [28, 29] and were actively concerned with preserving the dignity of the participants in the interviews and the subsequent research process.

## Result

We organized the findings into four main thematic categories, moving from parents’ experiences before receiving guidance to their initiations and the subsequent unfolding and transference of change processes to their everyday lives after the guidance, as follows: A) feeling inadequate or disconnected as a parent, B) discovering the infant as a relating and intentional person, C) becoming more agentic and interconnected and D) still feeling challenged by personal mental health issues. For confidentiality, all data are anonymized and all infants and parents are labelled as female.

### Feeling inadequate or disconnected as a parent

This first category describes the participants’ situation at the beginning of the guidance process. All participants were struggling with being parents, and feelings of loneliness and isolation were common to most of them. Many felt very insecure and inadequate as caregivers, and some felt emotionally disconnected. Many participants were parents for the first time and found the transition to parenting to be unsettling.

It was a bit scary … When I came home from the hospital, at first, I almost felt I was waiting for someone to - that I somehow had just borrowed the baby. That someone would come and take her away again. That I was a bit like a baby-sitter. I didn’t realize at first that she is actually here; this is my baby.

Parents who expected a high degree of perfection from themselves could feel overwhelmed in this new and uncharted territory and afraid to engage in caregiving activities. Even when they knew that their own standards and needs for control were impossible to meet, they still could not let them go: “I wanted to be perfect in a way, although I understand that nothing is perfect. One cannot be perfect, and I knew it then, and I know it now”. Some parents felt uncomfortable interacting with their infants. Holding or talking to their child did not feel natural and easy, and it could also be hard when the other parent seemed more competent and connected. This could enhance pre-existing doubts about being a good enough parent or being fit for parenthood at all, which, again, obstructed them from connecting with their child. Many parents described themselves as being prone to self-criticism. “I thought very negatively about myself in that role [as a parent], so I constantly found fault with myself.” Some experienced ambivalence about their perceived shortcomings: “There was actually a small voice inside me saying that what I did was right. But then somehow the negative thoughts entered and took over”.

Some parents described themselves as being removed from their feelings, as if their emotions were paralyzed. One parent exemplified this when relating how she missed noticing when her infant took her first steps:

*[direct speech is addressed at infant, who is not present during the interview:]*

“ … I didn’t even look up when you tried to make contact. It was as if I couldn’t manage then. I couldn’t manage to relate to your wanting to make contact.” So, I get a bad conscience - I get a bad conscience afterwards. That she took her first steps, and I wasn’t even looking.

Some parents experienced the absence of an emotional connection with the infant. They felt a lack of delight in the interaction that would have helped them create a sense of their belonging to the infant, or of the infant belonging to them: “I found it actually good to be away from the baby. I didn’t feel anything, and I could easily have stayed away for several days, the way I felt then.” This enhanced their experience of not coping, which again heightened a sense of emotional distance that could turn into more antagonistic feelings.

I did have very negative feelings about my baby before I came here. I did think in a way that she – destroyed my life, that she just - I thought about that - I did sort of look at her and think, “you actually destroyed my life.”

Another parent felt a connection in certain moments but found it difficult to sustain this. For her, pregnancy and birth had mainly felt like an extension of her newly built relationship with her partner, which was central for her own stability. Her infant’s presence intruded upon this relationship: “Now I am just ready for, ‘now it’s done, we’ve had this [the baby] together. Now I and [the baby’s father] will be alone and travel and so on.’ But then I realized, ‘sh*t, that’s not possible anymore.’” She described her child as obstructing her personal freedom and spontaneity, two essential parts of who she felt she was. For her, the main problem towards her infant was her lack of what she imagined to be “a mother’s feelings”, which would thwart her need to feel free. She compared herself with her best friend:

… she is just sort of, so absorbed. Like, from when [the friend’s baby] was born … She couldn’t even sleep because she … It was like she was in love. She just lay there and looked at her baby all night while it was sleeping. I just thought, “Jesus”. When [my infant] was born, I was, like, “Ok, can someone take the baby away now so I can sleep. I don’t feel like - I don’t feel like anything at all.”

For this mother, it was very difficult to find a viable mode of being a parent amidst her ideas of autonomy and self-abandonment. For most parents, their negative thoughts about themselves hindered them in their spontaneous interactions with their infants.

All parents found it especially challenging to handle an infant’s distress. They felt stressed and unable to understand what the infant was expressing and just wished that any distress might pass as soon as possible. They could also fear not being able to handle a tense situation: “I was afraid to go outside with her. A bit also, like, afraid of her. That she would not want to lie in her pram, or that she would suddenly wake up when I walked her in the forest”. To most, their infants’ crying signalled that they were not good enough and not coping effectively. Remaining emotionally present was demanding when the distress felt unbearable, or when they struggled to separate their infants’ feelings from their own. However, admitting to themselves that they needed help could take time:

When I first got the offer [for treatment], the baby was maybe four months old … and I thought, like, “hormones”, and “that’s how it is in the beginning”, but it never got any better. … I thought, “when I stop breastfeeding, everything will get much better”, but it didn’t. It just stayed the same, really. And when it somehow never got better … I thought, “No. Now I have to get help … I can’t live like this for, I don’t know, many years.”

### Discovering the infant as a relating and intentional person

The second category describes the initiation of changes in how the parents perceived their infants, their interactions and themselves. The change process began when they were shown minute details of their exchanges with their infants and discovered their infants’ contact-seeking behaviours. This touched them emotionally and made them more aware of their infant as a person. This, in turn, changed their experiences of connection, meaning and complexity in the interaction and of themselves as more secure and agentic parents.

Most parents described that it was their infant’s gaze that first captured their attention and engendered the change process. Discovering that their infant looked at them and followed them with her gaze was an unexpected experience that opened them up to the child. Initially, this discovery was mainly connected with themselves and how it made them feel more adequate, important or cherished as human beings and caregivers. Their infants’ contact initiatives and attempts to continue these connections arrested the parents and made them aware of how their infants sought them, waited for their attention and expressed delight in them.

I think it was when I saw it on video that the baby was very interested in what I did and what I said. That she smiled at me. That made me feel very good. I felt that she loved me, too, and not just [the other parent]. That I, too, was able to build up this connection. I guess this was what affected me … no matter what I did, she was interested. And, well - that I saw that gaze. The eye contact that the baby gave me.

Parents described this discovery as a repeated experience that gradually assured them of their importance to their infant and of being good enough parents: “She comes to me spontaneously, lies down in my lap or seeks physical contact …. That is very gratifying. And again and again reassures me that what I do is good enough.” Many parents related how they experienced the infant’s unwavering desire for contact as a feeling that they were intensely valued and cherished: “I was afraid that I couldn’t manage … But then I really saw that she adored me anyway.” One parent described how the repeated, reliable contact-seeking of her infant made her realize that she truly was of personal importance to her child, whereas before she had assumed it did not matter to her infant whether it was her or somebody else who was present. The infant’s gaze always returned to her, sought her, and rested upon her, which gradually made her trust that there was a special connection. Many parents described these gaze contacts and how their infants’ readiness to smile elicited joy in them. They felt more secure and confident in an infant’s presence and more competent as parents. This element in the change process then led to their being more interested in their infants.

… I [see] how much my baby looks up to me. She is following me all the time and wants my attention. And there is something in the gazes and expressions that you maybe don’t catch so easily while it’s happening. But when you see it from a little distance, you realize that, okay, maybe I actually have to begin to try to understand her a little better.

When parents increased their efforts to understand, their infants’ actions became more meaningful, which in turn made it easier for parents to figure out what was taking place in a particular interaction. They began to see more complexity in their exchanges with their infants and how their minute interactions influenced one another and were interconnected. This in turn made them view their infants more as complete persons and recognize their agency. Their infants’ behaviours were now seen as genuine communication. Parents experienced they could understand both their infants and themselves better and realized how much their own efforts at reading their infants’ signals meant.

I also saw how she appreciated - that she understood that I was trying to understand … I had never thought that … such a little baby could communicate so much without words. That was a shock for me.

Parents’ initial change processes unfolded from an increased awareness of their infants to a new perspective of their interactions and a changed experience of parenting. One parent described how the review sessions helped her to see that her infant was dependent upon her and loved her, and that this changed her own stance towards caring. Another parent related how her parental feelings were activated when she first became aware of her infant’s gaze initiatives: “that was a real eureka moment … just as if I could ‘see’ motherly love, you know?” Parents who had felt insecure and awkward about handling their babies gained more confidence when they realized that complex, prolonged exchanges were occurring between themselves and their infants and found it easier to submerge themselves in these interactions. This was experienced as a gradual, natural development.

### Becoming more agentic and interconnected

The third theme presents the further development of the change process. Parents described how they sustainably experienced themselves as more agentic and connected with their infants through video guidance. All parents said that the discoveries they made in the reviews also changed their perspectives on their daily interactions. Having first become more aware of the interactions made it easier to recognize the same exchanges in everyday life with a child and make them last longer.

It’s not so easy to be aware in everyday life … things go on autopilot all the time. But it has made me a little more aware that she is actually doing something, or looking at me in a certain way, so she wants to have contact. This makes it easier for me to recognize it, and then I can sit down alongside her and make something out of the situation instead of just thinking “Oh, hi” and then moving on to something else.

An important discovery for parents during reviews was to see how their actions and an infant’s actions were interconnected. Realizing this increased their own experiences of agency. Parents described how they slowed their pace and involved themselves more actively with their infants, even in stressful situations, because they now felt they had the ability to influence what was going on. They became more aware of how their own stress influenced their infants. “When I am stressed [ …] she becomes fussy and whiny. [Guidance] helped me to understand that this is interconnected. She is not being fussy for fun. I’m the one running around and being stressed out; no wonder it affects her.” This was a significant realization. First, their infants’ distress became more understandable. From experiencing their infants’ stress as uncomfortable, agitating or even antagonistic, parents began to see stress as a meaningful expression of needs and as a communicative act. In addition, the parents now also found it more meaningful to try to contain themselves in moments of stress. “I can now more easily calm myself down again and think, rationally, that I am good enough and I do what I can for the baby; I am aware of her.” These repeated experiences of being able to take greater charge when they remained aware and contained their own feelings of stress led to gradual changes of their parenting towards being more active and responsible.

If she is somehow angry or stressed, I think all the time, “okay, now at least I have to stay calm” so that she feels that I am relaxed and secure, and now we will find out what to do with this.

Parents developed more confidence, especially in demanding and tense situations. They described profound changes in being together with their infants and soothing them. Parents also experienced distress differently from a temporal perspective; things could be difficult in the present moment, but they would change again soon: “I try to just be patient and let her express herself, and try to figure it out, because it’s going to be alright [ …] it will end.” Furthermore, they also felt more stimulated to reflect upon the infants and the interactions because they found both to be more meaningful. They described a positive, self-enhancing circle of increased interest, increased understanding and increased experience of agency.

… Through Marte Meo, I have understood that I must try to read my baby. What is it that she is expressing? – and it doesn’t matter if that takes some time. The most important thing is that I try to understand … instead of panicking, “o, no, how terrible”.

Many parents described how they became more able to see an infant’s perspective and understand an infant’s signals. They discovered that a child expressed a range of feelings and communicated with them. Parents also recognized the developmental contexts of their infants: “It’s easier for me to understand why the baby is doing the things she does … related to her development. So, it doesn’t stress or irritate me so much in our daily life anymore”. When parents became aware of their infant as a meaningful person, they also saw more patterns and structure in the infant’s interactions; their infant became more predictable. Several parents related how these changes also led to seeing themselves as agentic, competent and connected parents. They described how the guidance gave them more security, which made them experience the bonds with their infants as being stronger but also more relaxed and realistic.

I am very attached to the baby, but in a more relaxed and balanced mode of thinking somehow - varying from day to day, actually. How much you feel you can manage … because one can have bad or good days.

Many parents found themselves more flexible and tolerant in everyday interactions with their infants. They had become less self-conscious in the interactions and often intuitively navigated the exchanges; “I’m different with her now [ …] more confident and sure about what I am doing”. Parents were able to give themselves more time to determine what their child expressed and felt more empowered to make decisions that they felt were right, even though these decisions might increase a child’s distress for a short time, such as putting an overtired baby to bed or prohibiting an unwanted activity. They were able to focus confidently on the resources they had rather than emphasise what they might lack. Thus, living with an infant became more complex, manageable and enjoyable.

I experience more pleasure and feel that pleasure, even though things can be tough sometimes. So, in a way, it has taught me to appreciate that I have the baby anyway and that I can understand her better. That there actually is a pleasure in that too … So, there is a change in that I feel a lot of pleasure. I love her very much.

Many parents described how they had developed a strong emotional connection and responsibility towards their children. They also narrated how their relationship with their infants became more personal and how they found their own individual roles as parents, which again strengthened their bonds and provided security. One parent described how she gradually dared to hope for change and trusted in her own adequacy. While she initially depended upon external confirmation, she developed an internal supportive voice that enabled her to remain connected with her infant where before she would have disconnected and used other activities to self-regulate.

[They] were good somehow, those moments when the thoughts - I could sit down on the sofa and cuddle. And play a bit, and then ask myself: “Could this be all that is needed?” That was actually a very good experience. Because there were no other thoughts. I forced myself in a way to focus just on the baby. “Don’t think, don’t think about the washing-up. Or checking the phone or stuff like that”. To look at her: “What is it that she wants?”

When the reviews showed how interactions changed, parents were enabled to believe in their transformations. One parent related her surprise about how relaxed and calm she now handled a situation of distress, and how this convinced her that she had integrated her discoveries from the previous review sessions. Many parents described how they had become more intuitive in their responses to their children.

In a way, it got easier when I saw how the baby reacted … gradually, just naturally, actually. Maybe now and then I would think I’d try, like, to say what we are about to do. Like “now we shall change the nappy”. Yet mainly I felt this just gradually came by itself. That when she was crying or something, I said something like, “oh, poor thing, does your tummy hurt?” Or, yes. I couldn’t do that before, so I just felt it developed. And now this just happens automatically.

These changes also affected other areas of everyday life. One parent narrated how she had gone from being generally pessimistic and expecting the worst to tackling challenges in an optimistic way: “Because I feel I have to be like that for her, or that it’s the best for both of us, I am automatically also like that in other situations in everyday life.” Another parent was able to be more open with her friends and family about her mental health issues and worries as a parent. She also stopped comparing herself to her partner, which led to better companionship around parenting issues and more openness in the relationship.

### Still feeling challenged by personal mental health issues

The fourth and final category describes the continuing emotional difficulties many parents experienced, despite also experiencing sustained and often profound changes. These difficulties were related to the parents’ psychological problems that still influenced their expectations or their perceptions, especially as parents. The psychological problems were felt also to be about parents themselves and not merely about external disorders: “I have said that I think this was never a postnatal depression. This is because I am as I am.”

Some parents still experienced the bond with an infant as weak or unstable. One described how she moved between feeling competent and optimistic and being ambivalent about her child and unsure of herself as a parent. The difficult feelings arose especially when her infant’s needs collided with her own needs or expectations, and when she could not handle distress. She would then collapse into feeling intensely frustrated, unloved and futile. Her unstable feelings conveyed to her that she lacked something essential needed for parenting.

It happens actually often at night that I am putting her to bed and ask for her forgiveness, and say “Sorry for being the worst mother in the world.” While she just [seems untroubled]. Yes, well – often actually with tears in my eyes. That, “Poor thing, you didn’t choose this. You could have had it much better, but this is how it went.”

Another parent still felt removed from both herself and her infant. While she now reflected more about her infant and herself and interacted with her child differently, she did not experience more feelings or a personal connection.

I do see she smiles. I see she has expectations, I see she seeks contact, I see that she gets happy … – I see all these things. It’s just that they - they don’t move anything inside me. It’s like watching whatever child on TV.

She described herself as being split into a sick part and a rational part, and she was afraid that she had used the guidance primarily as a technical tool for satisfying her infant’s needs so that she would be less overwhelmed by the infant’s demands. This increased her self-contempt and despair.

However, even though most of the other parents saw their persisting tendencies towards insecurity and self-doubt as a part of who they were as persons, they now felt more secure and competent as parents, and said that the experiences from the guidance still lingered and nourished their self-esteem. Their form still varied on certain days. What had changed, however, was their way of understanding and handling these variances.

I have become more secure about myself and I understand the baby better … Back then, I mostly became irritated when she cried a lot, and I couldn’t understand that this actually is the way she communicates … But one should bear in mind how it is when one is tired or needs attention … because I also have days when I am not super stable. When I also need that little extra something. … It helps that I also manage to see it from this perspective.

Many parents related that it was now less important whether they still had problematic or unresolved mental health issues. Even though the interactions could feel rocky at times, now parents mostly felt that they could cope with their infants: “I am not fully cured, that has to be said. But … now I have learnt to accept recognition for the job I am doing as a parent … no matter if I am pulling my hair in frustration now and then.” One parent described how she still struggled and doubted herself, but now she felt agentic:

I know many people are better than me in many things, so I have to be strong enough to ask … “Can you teach me that?” I want to become good, too … I am very open towards new things. Not everything has to be an obstacle. It can be a challenge, and so I take up the challenge. And I do my best, because then I have tried. If I fail, well, so I failed, but I tried. I did something about it. I am not left with the feeling that “why didn’t I do something about that?” That I always try as best as I can.

Most parents adjusted their outlooks towards more realistic and hopeful perspectives on parenting and on their own abilities. Their own insecurities were no longer experienced as hindrance to being a parent, but rather as parts of who they were in their everyday lives.

## Discussion

In our analysis of how parents who had difficulties mentalizing and remaining emotionally connected with their infants described their own experiences of change during and after Marte Meo video guidance, we identified four main categories. In the following, we discuss the results in light of established perspectives, with the aim of further developing the existing conceptual understandings of Marte Meo and its clinical uses with these clients.

The first category, “Feeling inadequate or disconnected as a parent”, shows how the predominance of preoccupied and self-critical states may have hindered parents’ normal development of caregiving through repeated successful interactions with their infants [30, 31]. An inhibition to touch, handle and vocalize with an infant points to difficulties in a parent’s intuitive parenting capacity [32, 33]. Parents’ tendencies to not cohesively regard infants as relational and intentional beings and to read infant cues as negative references about their own caregiving, which represent a low degree of mentalizing, may have maintained negative transactional feedback loops [34]. This is in line with evidence that parents with mental health problems experience more early feelings of estrangement from, anxiety towards and anger with their infants [35, 36], constituting risk factors for infant development [37] and contributing to manifest relationship disturbances [38]. Therapists working with insecure parents have described how they shift parents’ awareness from themselves onto their infants and into the present, focusing especially on contact moments [39]. The visual focus of guidance on successful, minute interactions and contact moments to highlight existing resources in both parents and infants to support intuitive caregiving competence [34] may be especially suited for this parent group.

The second category, “Discovering the infant as a relating and intentional person”, depicts the incipient change process that is sparked by a parent’s surprise upon discovering an infant’s relatedness. However, parents’ initial reading of this discovery was still centred on what this relatedness meant in terms of their own lovability, adequacy, and importance. Gill’s study of a specialized setting mentions a similar self-centred reading of the child’s gaze [10]. The surprise, or “shock”, on seeing videos of their interactions has been hypothesized to be facilitating access to parents’ unconscious material relating to their own attachment and stimulating a new organization and reflection [4, 40]. The findings from our parent group indicates that this surprise first jolted their preoccupations with self-conscious emotions [41] and supported changes towards better acceptance of positive relational experiences that were directed at themselves. This process seemed to precede and engender their increased engagement, joy, and experience of mutual connectedness and competence, which in turn formed the basis for their mentalizing stance.

The third category, “Becoming more agentic and interconnected”, shows the integration of new relational experiences in everyday life with an infant. Increased self-regulation and tolerance in the face of infant distress and description of distress as now having a temporal structure suggest a change at the representational level of negative emotions, becoming symbolized, second-degree representations [42]. Infant distress was now also experienced as a communicative cue, eliciting an intuitive caregiving response [43], affect regulation and marked mirroring [44]. These positive reinforcing transactional circles [34] enhanced parents’ representations of themselves as competent caregivers [31]. Parents reported key facets of increased mentalizing in their interactions with their infants, such as their sustained curiosity about the infants, their awareness of their infants’ and their own mental states being complexly interconnected; their focus on affect-regulating the infants, their considering developmental perspectives in the interactions [45]; and their descriptions of increased self-regulation and mentalization in functional areas other than caregiving.

The final category, “Still feeling challenged by personal mental health issues,” emphasizes how change is often an ongoing process embedded in a parent’s psychosocial situation. Most parents integrated more flexible ideas of themselves as caregivers, with strengthened experiences of connection and self-confidence, even amidst challenges. This suggests a fluid shifting between representations, between past and present, and between self and infant [31]. Parents were better able to navigate conflicts, which can be understood to result from increased relational security [46]. Their view of everyday life with their infants was more relaxed and balanced and thus more normalized, as ambivalence and insecurity are common fleeting states in parenting [31]. Their confidence expanded to not only other situations and relationships but also how they handled persisting mental health problems, which most parents still experienced with varying frequency. However, for some parents, these problems remained very burdensome, indicating more deeply engrained mental health challenges that may necessitate a better coordination of interaction guidance with a parent’s primary therapeutic process.

Overall, these findings indicate that Marte Meo guidance supported crucial changes amongst this strategic sample of parents. The change process seemed to be sparked by parents feeling sufficiently encouraged to emotionally connect with the actual interactions, as presented in the video clips. Feeling loved and validated by their infants’ contact-seeking behaviour seemed to supply this encouragement, especially when this behaviour came as a surprise, with the ability to jolt pre-existing ideas or schemata. Visual encounters with infants’ benign nature may have helped parents to discriminate between their actual infants and their internal representations of earlier, less benign relational experiences [46, 47] to facilitate relational openings towards their children. This “discovery” of their actual infants led to more positive emotions, experiences of reciprocal connectedness, and interest in their children, supporting further developments in two areas; namely, in mentalizing and in experiencing themselves as agentic and influential. Regarding VIG, a video guidance method with similarities to Marte Meo [48], conceptual papers have discussed the discrepancies between parents’ old, negative beliefs about themselves and the evidence of positive interactions presented in video feedback, which have been posited to create a cognitive dissonance that promotes metacognitive capacity, or mentalization, as well as increased self-efficacy and empowerment [49, 50]. In an analysis of Marte Meo method elements, the combination of unreality or distance created by video and the experience of film as concrete proof has been identified as a relevant facilitating factor (Simhan I, Vik K, Veseth M, Hjeltnes A: Like taking a magnifying glass into everyday life: vulnerable parents’ experiences with video guidance in an infant mental health clinic, submitted for publication.). The experiences of surprise presented in this paper seemed to capture aspects of the change process that pertain more to overcoming avoidance, or the fear, of relationally connecting, as a first step towards an increased mentalizing capacity. This opening towards more positive relational experiences and increased connections with the visually presented interaction were central elements that supported the increases in reflective functioning and sensitivity. After guidance, most parents were able to more flexibly shift between positive and negative states and to represent their negative emotions and distress on a symbolic level. Our findings imply that the guidance stimulated change, both on an interactional and representational level and in parents’ reflective functioning. The presence of the attuned therapists who regulated the parents and invited them to reflection is assumed to be an important factor that facilitated these relational changes and increased mentalization capacities [9, 49]. Parents also described how the therapists’ presence lingered with them even after guidance sessions and promoted an integration of their new experiences (Simhan I, Vik K, Veseth M, Hjeltnes A: Like taking a magnifying glass into everyday life: vulnerable parents’ experiences with video guidance in an infant mental health clinic, submitted for publication.).

Most parents experienced increased agency, flexibility and confidence as parents and in other areas of their lives, including their own mental health challenges. This seems to reflect a pivotal increase in a parent’s experience of self-efficacy [51], not only in the domain of parenting but also on a general level. Parents’ self-efficacy is defined as “confidence about their ability to successfully raise children” and is linked to several positive parenting functions. General self-efficacy more broadly involves positive self-perceptions of one’s agency, competency and influence over events [52, 53]. Enhanced self-efficacy in this sample of parents promoted relatedness, affect regulation, mentalization and intuitive parenting competency to extend more generally to coping with mental health issues. These findings relate well to knowledge from the recovery movement in mental health, which has repeatedly demonstrated how people are able to find ways of leading meaningful lives in the face of a wide range of different mental illnesses [54]. Davidson et al. have defined recovery as “a process of restoring a meaningful sense of belonging to one’s community and positive sense of identity apart from one’s condition while rebuilding a life despite or within the limitations imposed by that condition” [55]. This strengthens the suggestion of earlier conceptualizations that have placed Marte Meo amongst resource-oriented methods [8] and aligns with research that has demonstrated symptom reduction in depressed parents from a community sample [7].

### Reflexivity and methodical considerations

Reflexivity involves systematic efforts to articulate how researchers’ own preconceptions and subjectivity might inform and influence how data are acquired, analysed, organized and interpreted [19, 20, 56]. Aiming at an experience-near analysis of parents’ experiences demanded reflexivity from the research team. The first author, an infant psychiatrist with a background in video interaction guidance, derived her interest in the parents’ lifeworld from her clinical work. This had the potential to impose preconceptions derived from her own experiences with parent-infant dyads. Therefore, conceptualizing, including conceptual language, was consciously set aside during data analysis. Moreover, the analysis process was critically moderated by the fourth author and audited by the third author. Both had experience with therapeutic processes but not with infant mental health or video guidance. The second author was one of four Marte Meo therapists providing the interventions. She took part in the development of the study design, the discussion of the results, and the conceptualization but not in the data analysis.

### Limitations

The present study has several methodological limitations. Our participants, while all recruited from a specialist clinic and striving to mentalize and connect emotionally with their infants, were heterogeneous regarding their self-reported mental health issues. Our findings do not allow for discrimination between different mental conditions. Moreover, transferring our results to other parent or patient populations should be done with care. It could be argued that the narrative interview we employed in the sampling process mainly addresses mentalization in speech, neglecting its embodied manifestations [57]. Our qualitative approach investigated the parents’ subjective experiences of Marte Meo video guidance. It cannot determine causal relations between the described phenomena. The interviews varied in length, which may have led to more dismissing parents having less prominent voices in the data. Among our participants, women were overrepresented, with the risk of a skewed description of parenting experiences.

### Implications for research and clinical practice

The findings in this study have several important implications for research and clinical practice. As they suggest that video guidance using the Marte Meo method can stimulate a profound change in dyads that have been referred to specialized treatment, research should investigate the application of the method for defined parental mental health issues, such as personality disorders, recurrent depression, substance misuse or posttraumatic disorders. These investigations should include changes in mentalization capacity and self-efficacy. An interesting topic would be a combination of parental video guidance and parents’ primary therapeutic treatment. Additionally, more research on fathers as a parent group would be useful. Moreover, future research should cover parents of older children with more deeply engrained patterns of interacting and a more developed capacity for verbal communication to identify whether their change processes differ from those of parents of infants.

For clinical work, our findings suggest that Marte Meo can be useful in the specialized treatment of dyads where parents have complex psychosocial or mental health issues and difficulties mentalizing their infants. This study’s parents’ experiences indicate that there are concrete requirements for video guidance. Therapists should be aware that, initially, parents may be very preoccupied with self-conscious emotions. To initiate change, the review sessions should focus on showing an infant’s gaze and contact initiatives. Especially for insecure parents, these moments should be shown repeatedly. Once parents become engaged, film clips showing mutuality and interconnectedness in interactions with an infant can stimulate their further awareness and reflection. As the parents’ own challenges and parenting seem closely interconnected, and as the experience of agency and confidence seem to be central focal points, a combination of guidance with the parents’ primary treatment could be considered. Our findings imply that these change processes involve change at the levels of both interaction and representation. Guidance can strengthen vulnerable parents’ connectedness, mentalization and self-regulation, as well as their self-efficacy, both as parents and in other areas of life.

## Conclusion

The exploration of vulnerable parents’ experiences of change during Marte Meo video guidance elicited several aspects of the therapeutic process. At the outset, many parents felt disconnected, could not engage their intuitive parenting capacities, and interpreted their infants’ cues as negative feedback. The change process was engendered when they discovered their infants’ relational interest, which at first centrally confirmed and strengthened their self-worth and ability to relate. This led to a cascade of increased engagement and joy and an experience of mutual connectedness and competence, which in turn formed the basis for a mentalizing stance and reflective functioning. Changes occurred at the interactional and representational levels and through increases in parents’ self-efficacy, flexibility and confidence, not only as caregivers but also in other relationships and towards their persisting mental health issues. The findings suggest that video guidance can be a critical intervention for parents who struggle to mentalize and maintain an emotional connection with their infants, but it should be coordinated with primary treatment when complex parental mental health issues are involved.

## Data Availability

The qualitative datasets generated and/or analysed during the current study are not publicly available due to reasons of confidentiality but are available from the corresponding author on reasonable request (Norwegian only).

## Abbreviations

PDI: Parent development interview
IMHT: Infant mental health team
RF: Reflective functioning
TA: Thematic analysis
VIG: Video interaction guidance
VIPI: Video-guidance of parent-infant interaction
